# Sexually dimorphic adaptation of cardiac function: roles of epoxyeicosatrienoic acid and peroxisome proliferator‐activated receptors

**DOI:** 10.14814/phy2.12838

**Published:** 2016-06-28

**Authors:** Jun Qin, Yicong Le, Ghezal Froogh, Sharath Kandhi, Houli Jiang, Meng Luo, Dong Sun, An Huang

**Affiliations:** ^1^ Department of PhysiologyNew York Medical CollegeValhallaNew York; ^2^ Department of GI SurgeryRenji HospitalSchool of MedicineShanghai Jiaotong UniversityShanghaiChina; ^3^ Department of PharmacologyNew York Medical CollegeValhallaNew York; ^4^ Department of GI SurgeryShanghai Ninth People's HospitalSchool of MedicineShanghai Jiaotong UniversityShanghaiChina

**Keywords:** Cardiac contractility, epoxyeicosatrienoic acids, peroxisome proliferator‐activated receptors, sex, soluble epoxide hydrolase

## Abstract

Epoxyeicosatrienoic acids (EETs) are cardioprotective mediators metabolized by soluble epoxide hydrolase (sEH) to form corresponding diols (DHETs). As a sex‐susceptible target, sEH is involved in the sexually dimorphic regulation of cardiovascular function. Thus, we hypothesized that the female sex favors EET‐mediated potentiation of cardiac function via downregulation of sEH expression, followed by upregulation of peroxisome proliferator‐activated receptors (PPARs). Hearts were isolated from male (M) and female (F) wild‐type (WT) and sEH‐KO mice, and perfused with constant flow at different preloads. Basal coronary flow required to maintain the perfusion pressure at 100 mmHg was significantly greater in females than males, and sEH‐KO than WT mice. All hearts displayed a dose‐dependent decrease in coronary resistance and increase in cardiac contractility, represented as developed tension in response to increases in preload. These responses were also significantly greater in females than males, and sEH‐KO than WT. 14,15‐EEZE abolished the sex‐induced (F vs. M) and transgenic model‐dependent (KO vs. WT) differences in the cardiac contractility, confirming an EET‐driven response. Compared with M‐WT controls, F‐WT hearts expressed downregulation of sEH, associated with increased EETs and reduced DHETs, a pattern comparable to that observed in sEH‐KO hearts. Coincidentally, F‐WT and sEH‐KO hearts exhibited increased PPAR *α* expression, but comparable expression of eNOS, PPAR *β*, and EET synthases. In conclusion, female‐specific downregulation of sEH initiates an EET‐dependent adaptation of cardiac function, characterized by increased coronary flow via reduction in vascular resistance, and promotion of cardiac contractility, a response that could be further intensified by PPAR *α*.

## Introduction

The incidence of ischemic heart diseases distributes in a sexually dimorphic manner, as evidenced by the significantly lower incidence in females than age‐matched males, which is due at least in part, to the better cardiac perfusion in women (Gura 1995; Huang and Kaley 2004). A potential gene involved in the sexual dimorphism of cardiovascular diseases is the Ephx2 that encodes for the protein of soluble epoxide hydrolase (sEH) and is responsible for converting cardioprotective mediators, namely epoxyeicosatrienoic acids (EETs; members of epoxy fatty acid family), to their corresponding non‐ or less bioactive diols (dihydroxyeicosatrienoic acids, DHETs) (Kersten et al. 2000; Mark‐Kappeler et al. 2011; Harris and Hammock 2013). Therefore, sEH is a susceptible gene for hypertension‐associated cardiovascular diseases (Kersten et al. 2000; Ai et al. 2007). A review of the literature shows that potentiation of EET bioavailability through compromising EET degradation, via either knockout of the Ephx2 gene or by pharmacological inhibition of the enzyme, evokes cardiovascular protective actions (Qin et al. 2015a,b). However, the nature of disruption of the Ephx2 gene affecting blood pressure (Luria et al. 2007) and vascular responsiveness (Kandhi et al. 2015b; Qin et al. 2015a) in a sex‐different manner remains to be clarified. Findings of sexual dimorphism in the regulation of sEH expression/or activity were originally reported in the 1980s, when the results were exclusively obtained from the liver and kidney of rodents, but reported without functional relevance (Gill and Hammock 1980; Meijer et al. 1987; Denlinger and Vesell 1989; Pinot et al. 1995). Only currently has the sexually dimorphic regulation of sEH come to the forefront of research to specifically address sEH‐dependent detrimental actions in the pathogenesis of cardiovascular diseases. Later studies by Alkayed's group revealed a sex‐specific phenotype, characterized as a female/estrogen‐dependent suppression of sEH in the cerebral circulation, a mechanism that is believed to be responsible for the female‐favorable protection from cerebral ischemic damages such as stroke (Koerner et al. 2008; Fairbanks et al. 2012; Davis et al. 2013; Zuloaga et al. 2014). It is worth noting that the aforementioned studies regarding the sexually dimorphic regulation of sEH in the cerebral circulation were conducted exclusively on pathological models, such as cerebral artery occlusion, or exposure of cultured neuronal and cerebral artery endothelial cells to oxygen–glucose deprivation. Therefore, research aiming to determine the physiological significance of sex‐differential regulation of sEH has been left unaddressed. To date, we have provided evidence indicating that female‐specific downregulation of sEH expression in coronary, mesenteric, and skeletal muscle arterioles contributes significantly to the attenuation of pressure‐induced coronary vasoconstriction (Froogh et al. 2016) and potentiation of shear stress‐induced vasodilation (Qin et al. 2015a). While we have also demonstrated that genetic deletion or pharmacological inhibition of sEH enhances coronary perfusion to improve cardiac function (Qin et al. 2015b), the specific role of sex in the physiologically control of cardiac function via EET/sEH signaling remains unknown.

On the other hand, highly expressed peroxisome proliferator‐activated receptor‐*α* (PPAR*α*) in the myocardium has been demonstrated to serve as an essential player in the regulation of cardiac oxidation of fatty acids (Desvergne et al. 2006), which are a major energy source necessary for heart contractility (Lopaschuk et al. 1994). PPARs are transcription factors that regulate and target gene transcription via a ligand‐dependent reporter binding, which provides the molecular basis for an EET‐attributable activation of PPARs through their actions as PPAR ligands (Kersten et al. 2000). Within the signaling networks of fatty acid metabolism via estrogen/CYP/EETs/sEH, PPARs serve as the central mediator either as a downstream target of EETs or an upstream initiator of estrogen/CYP (Imig et al. 2005) actions. Indeed, the crucial role of PPAR*α* in the conduction of cardiac metabolic regulatory programs has been well documented (Burkart et al. 2007). Based on evidence that sEH can be targeted in a sexually dimorphic manner and activation of PPAR signaling is linked to EET bioactivity (Kersten et al. 2000; Harris and Hammock 2013), we hypothesized that the female sex favors the EET‐dependent adaptation of cardiac function via downregulation of sEH expression to increase cardiac EET bioavailability, the response that might be in turn enhanced by reciprocal activation of PPAR signaling.

## Materials and Methods

### Animals

Twelve‐ to fifteen‐week‐old male (M) and female (F) Ephx2^−/−^ (sEH‐KO) and wild‐type (WT) mice, abbreviated as M‐KO, F‐KO, M‐WT, and F‐WT, respectively, were used. As described previously (Sun et al. 2014; Qin et al. 2015b), cryorecovered heterozygous (Ephx2^+/−^, B6.129X‐Ephx2tm1Gonz/J) and WT (Ephx2^+/+^, C57BL/6J) mice were received from Jackson laboratory (Bar Harbor, ME) and the homozygous (Ephx2^−/−^) sEH‐KO mice were developed in the Department of Comparative Medicine, New York Medical College.

All protocols were approved by the Institutional Animal Care and Use Committee of New York Medical College and conform to the guidelines of the National Institutes of Health and the American Physiological Society for the use and care of laboratory animals.

### Measurement of blood pressure

Mice were anesthetized by inhalation of Isothesia (isoflurane). Blood pressure was recorded using carotid artery catheterization and a flow‐through pressure transducer (TRN050; Kent Scientific, Torrington, CT). The heart rate of anesthetized mice was controlled at approximately 480 beats per minute by adjusting the depth of anesthesia when the blood pressure was measured (Kandhi et al. 2015b; Qin et al. 2015a,b).

### Isolated perfused hearts

As described previously (Qin et al. 2015b), mice were anesthetized with isoflurane, and heparin (100U) was injected intraperitoneally. The thorax was then opened. The heart was excised and immediately mounted via the ascending aorta onto a perfusion apparatus. The heart was perfused with a nonrecirculating physiological salt solution (PSS) containing (in mmol/L) NaCl 117, KCl 4.7, MgSO_4_ 1.1, KH_2_PO_4_ 1.2, glucose 5.5, CaCl_2_ 2.5, pyruvate 2.0, ascorbate 0.1, l‐arginine 0.1, and NaHCO_3_ 24. The PSS was gassed in a water‐jacketed (37°C) reservoir with 95% O_2_ to 5% CO_2_ to a pH value of 7.4. The perfusate was pumped through an in‐line 1‐*μ*m filter using a Rainin peristaltic pump. A 2‐mL bubble trap, a water‐jacketed (37°C) heating coil, and a T‐adaptor connecting a pressure transducer (P23XL, Grass) were sequentially connected to the heart. A metal hook made from a 31‐gauge needle was penetrated through the left ventricle at the apex of the heart to drain the Thebesian fluid accumulated in the ventricle. The other end of the hook was attached to a force transducer (FTO3C, Grass) to control and record tension and heart rate. The heart was immersed in the coronary effluent solution in a water‐jacketed (37°C) tissue bath (10 mL, Radnoti) and perfused with a constant flow. Changes in perfusion pressure and end‐diastolic and peak systolic tensions were continuously recorded. At the beginning of experiments, the flow rate was rapidly increased to reach a perfusion pressure of 100 mmHg. During the equilibration, changes (increases) in perfusion pressure as a consequence of development of myogenic constriction in coronary arteries were continuously adjusted by reducing flow rate to maintain a perfusate pressure at 100 mmHg. The heart reached an equilibrated status (a constant perfusion pressure of 100 mmHg and a constant heart rate of above 320 beats per min) within 15 min. The flow rate provided to obtain a constant perfusion pressure of 100 mmHg was considered as basal coronary flow and was kept constant throughout experiments. After basal flow was obtained, tension was increased from 0 to 4 g in 0.5 g increments to initiate a stepwise increase in end‐diastolic tension of the heart. At each tension step, changes in perfusion pressure were continuously recorded for 5 min on a DI‐700 data acquisition system (DATAQ).

In separate groups of mice, 14,15‐epoxyeicosa‐5(Z)‐enoic acid (14,15‐EEZE, 10^−5^mol/L; a putative pan‐EET receptor antagonist) was added into the PSS. Hearts were perfused with inhibitor‐containing PSS for 45 min and then changes in perfusion pressure/developed tension in responses to stepwise increases in diastolic tension were recorded.

### LC/MS/MS‐based measurements for cardiac EETs and DHETs

Isolated hearts were pulverized in liquid nitrogen. Phospholipids were extracted with the Bligh–Dyer method from 30 to 50 mg of heart tissue. EETs and DHETs were extracted with ethyl acetate following alkali hydrolysis of the phospholipids to release esterified EETs or DHETs and quantified with a Q‐trap 3200 linear ion trap quadruple LC/MS/MS (AB ScieX; Qtrap 3200) equipped with a Turbo V ion source operated in negative electrospray mode (Applied Biosystems, Foster City, CA), as described previously (Sun et al. 2014; Kandhi et al. 2015b; Qin et al. 2015a,b). Protein concentration of samples was determined by the Bradford method (Bio‐Rad, Hercules, CA) and was used to normalize the detected lipids. Data for total EET and DHET levels, as well as for each of the four regioisomeric EETs and DHETs are presented and expressed as pg/mg protein.

### Western blot analysis

Isolated hearts were pulverized in liquid N_2_. Equal amounts of total protein (25 *μ*g) extracted from hearts were loaded onto and separated by a 10% SDS‐PAGE gel and transferred to a PVDF membrane. The membrane was probed with specific primary antibodies for sEH (1:800 dilution), PPAR*α* and PPAR*β* (1:500), eNOS (1:200), CYP2J2 (1:800) (Santa Cruz Biotechnology, Inc., Paso Robles, CA) and CYP2C9 (1:1000; Biodesign Inc. Maco, ME), and appropriate secondary antibodies conjugated with horseradish peroxidase (1:5000). Specific bands were visualized with a chemiluminescence kit (Thermo Scientific, Rockford, IL) and normalized to GAPDH. The X‐ray film was scanned into a computer and band densitometry was digitalized with UN‐SCAN‐IT software (Silk Scientific, Orem, UT).

### Calculation and statistical analysis

Data are expressed as mean ± SE, and *n* refers to the number of mice. Basal coronary flow was recorded at a constant pressure of 100 mmHg and normalized to the heart weight, and expressed as mL/min/g. Coronary vascular resistance was calculated by the formula of *R* = *P*/*F*, where *P* and *F* are indicative of perfusion pressure and flow, respectively, and expressed as mmHg/min per mL. Analyses of linear regression and the comparison of the slope of regression lines between multiple groups were performed using GraphPad Prism software (GraphPad Software, Inc., La Jolia, CA). Analyses of blood pressure, cardiac EET and DHET levels, and coronary basal flow were performed using one‐way ANOVA followed by the Tukey–Kramer post hoc test. Student's *t*‐test or paired *t*‐test was used where appropriate. Statistical significance was accepted at a level of *P* < 0.05.

## Results

### Reduced blood pressure in female WT and sEH‐deficient mice

Mouse mean arterial blood pressure (MAP) was recorded: M‐WT (*n* = 6): 93.5 ± 0.4 mmHg; F‐WT (*n* = 8): 84.5 ± 0.5 mmHg; M‐KO (*n* = 6): 83.2 ± 0.5 mmHg; and F‐KO (*n* = 6): 78.5 ± 1.0 mmHg. Female mice (F‐WT and F‐KO) had significantly lower MAP than their male (M‐WT and M‐KO) counterparts (*P* < 0.05). Compared to M‐WT mice, deletion of the sEH gene (M‐KO) significantly reduced blood pressure (*P* < 0.05) to a level comparable to that of F‐WT mice. F‐KO mice had the lowest MAP among the four groups of mice. The results revealed that the female sex potentiated sEH deficiency‐induced reductions in blood pressure.

### Sex‐different regulation of cardiac EET metabolism

**Figure 1:**
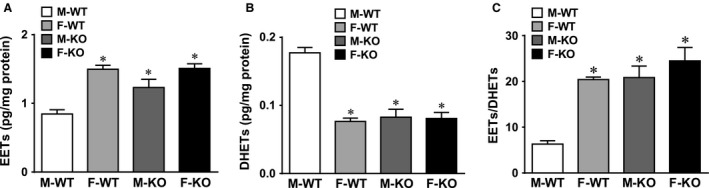
Increased EETs and reduced DHETs in myocardium of sEH‐KO and female WT mice. Normalized data for total levels of (A) EETs, (B) DHETs, and (C) the ratio of EETs/DHETs in myocardium of male (M) and female (F) WT and sEH‐KO mice. * indicates significant difference from M‐WT (*n* = 5–9). sEH, soluble epoxide hydrolase; EETs, epoxyeicosatrienoic acids; DHETs, dihydroxyeicosatrienoic acids; WT, wild type; KO, knockout.

Table 1 summarizes each individual EET and DHET regioisomer in the myocardium of the four groups of mice. The total levels of EETs and DHETs, as well as the ratio of EETs/DHETs are depicted in Figure 1. Figure 1 indicates several points: (1) M‐KO mice exhibited a significant increase in total EETs (A) as a consequence of decreased conversion of EETs into DHETs (B), leading to a greater ratio of EETs/DHETs compared to M‐WT controls (C), thereby confirming the disruption of the Ephx2 gene. (2) Compared to M‐WT, F‐WT mice exhibited a significantly greater ratio of EETs/DHETs as a result of increased EETs and decreased DHETs, suggesting a sex‐different regulation of EET metabolism in normal hearts. (3) A sex‐different response to sEH deficiency was specifically indicated by the fact that F‐KO mice did not initiate statistically significant changes in cardiac EET/DHET profile in comparison with F‐WT controls, while significance was observed in the cardiac EET/DHET ratio of M‐KO mice compared to their M‐WT mice. (4) The identical pattern of cardiac EET metabolism in F‐WT, F‐KO, and M‐KO mice implies the presence of redundant/or overlapping regulatory mechanism(s) between female sex and sEH deficiency.

**Table 1:** Profile of each regioisomeric epoxyeicosatrienoic acid (EET) and dihydroxyeicosatrienoic acid (DHET) in the myocardium of mice

	14,15‐EET	11,12‐EET	8,9‐EET	5,6‐EET	14,15‐DHET	11,12‐DHET	8,9‐DHET	5,6,‐DHET
M‐WT (*n* = 9)	0.149 ± 0.033	0.246 ± 0.063	0.279 ± 0.084	0.171 ± 0.029	0.032 ± 0.009	0.027 ± 0.010	0.067 ± 0.008	0.051 ± 0.015
F‐WT (*n* = 6)	0.257 ± 0.029	0.40 ± 0.054	0.392 ± 0.051	0.447 ± 0.038	0.011 ± 0.003	0.014 ± 0.002	0.030 ± 0.003	0.020 ± 0.005
M‐KO (*n* = 5)	0.217 ± 0.034	0.287 ± 0.057	0.388 ± 0.148	0.338 ± 0.058	0.011 ± 0.004	0.007 ± 0.003	0.025 ± 0.007	0.041 ± 0.018
F‐KO (*n* = 5)	0.302 ± 0.023	0.458 ± 0.063	0.359 ± 0.081	0.390 ± 0.029	0.010 ± 0.004	0.015 ± 0.005	0.031 ± 0.012	0.024 ± 0.009

### Female sex and sEH deficiency increase basal coronary flow

**Figure 2:**
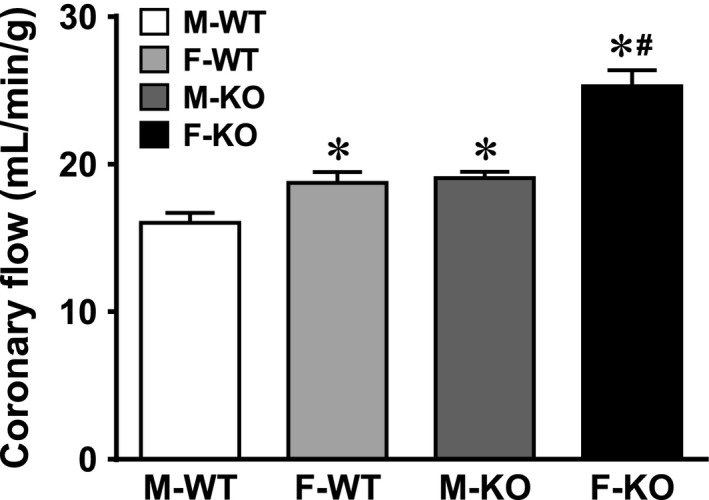
Increased basal coronary blood flow in sEH‐KO and female WT mice. Normalized data for basal coronary flow obtained at zero preload, from M‐WT (*n* = 8), F‐WT (*n* = 7), M‐KO (*n* = 7), and F‐KO (*n* = 6) mice, respectively. * indicates a significant difference from M‐WT. # indicates a significant difference from F‐WT and M‐KO, respectively.

Figure 2 summarizes data of basal coronary flow normalized to heart weight, revealing that in order to maintain a constant perfusion pressure of 100 mmHg, a significantly greater basal coronary flow was required in F‐WT than M‐WT mice, indicating a sex difference in the physiological control of coronary circulation. Compared to WT controls, sEH‐KO hearts required further increases in basal flow to maintain the perfusion pressure at 100 mmHg, and this increment exhibited a greater predominance in F‐KO than M‐KO mice. These results indicate that sEH‐deficiency initiates an increase in basal coronary flow, a response that is in a female‐favorable manner.

### Female sex favors sEH deficiency‐induced potentiation of cardiac work

**Figure 3:**
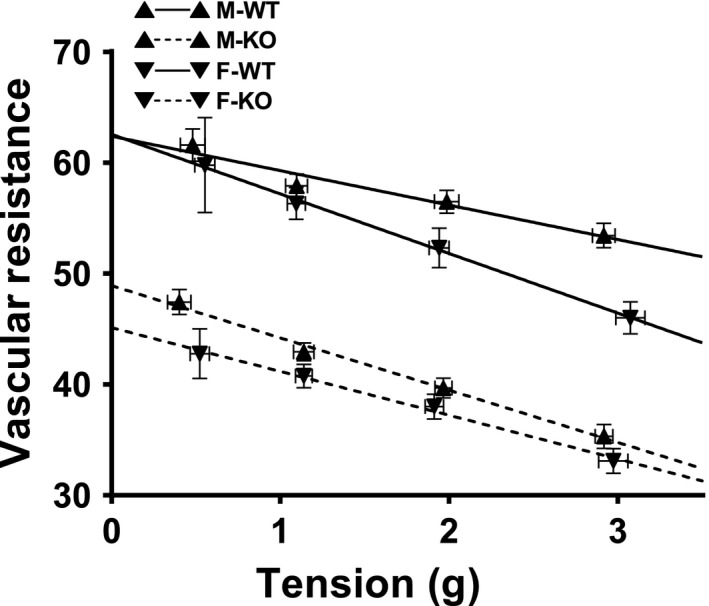
Attenuation of coronary resistance in sEH‐KO and female WT mice. The correlation between coronary vascular resistance and end‐diastolic tension in isolated hearts of M‐WT, F‐WT, M‐KO, and F‐KO mice. Correlation coefficients in the four groups of mice are −0.9748 (*P* < 0.05; M‐WT), −0.9999 (*P* < 0.01; F‐WT), −0.9965 (*P* < 0.01; M‐KO), and −0.9965 (*P* < 0.01; F‐KO), associated with the slope of their regression lines of −3.108 ± 0.5026, −5.374 ± 0.1669, −4.715 ± 0.2795, and −3.951 ± 0.2334, respectively. The difference in the slope between M‐WT and F‐WT, as well as M‐WT versus M‐KO, is statistically significant (*P* = 0.0123 and *P* = 0.04842, respectively) (*n* = 6–8 for each group).

Changes in perfusion pressure, expressed as changes in vascular resistance (*y*‐axis), were recorded in response to stepwise increases in end‐diastolic tension (*x*‐axis) in hearts. As depicted in Figure 3, coronary resistance at zero tension was inversely proportional to the level of basal perfusion flow shown in Figure 2. Increases in end‐diastolic tension dose dependently reduced vascular resistance in all groups of hearts. F‐WT hearts displayed a significantly greater magnitude of reduction at each preload, as well as at the rate of reduction, which was manifested by a deeper slope of the regression line compared to M‐WT hearts (*P* = 0.0123). This suggests a female‐favorable modulation of coronary resistance during cardiac work. Moreover, deletion of the sEH gene initiated a significantly drastic reduction in coronary resistance in both male and female (M‐KO and F‐KO) hearts compared to their corresponding WT hearts. These results imply that sEH deficiency potentiates coronary vasodilatation, via perhaps, an EET‐mediated mechanism. It is important to note that the sex‐difference in the reduction of coronary resistance was not statistically significant between M‐KO and F‐KO mice, nor was the slope of their regression lines (*P* = 0.1046). In this context, the lack of significant changes between male and female sEH‐KO mice suggests that the contribution of EETs to the response is essential, which was further supported by an identical cardiac EET metabolic profile between M‐KO and F‐KO mice (Fig. 1).

**Figure 4:**
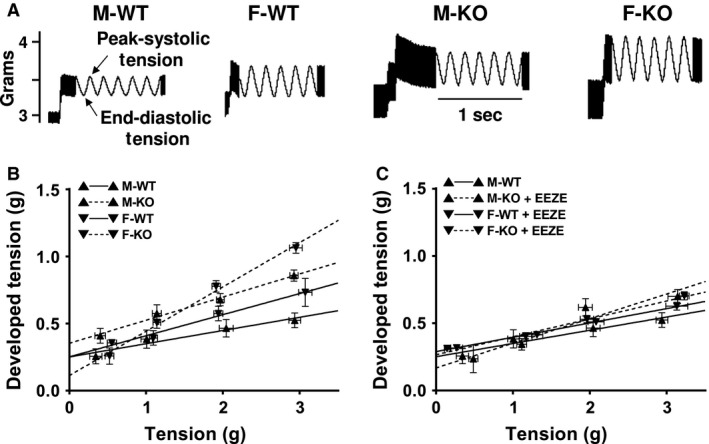
EET‐dependent promotion of cardiac contractility in sEH‐KO and female WT mice. (A) Tracings of heart contraction (peak systolic tension) in response to 0.5 g end‐diastolic tension in M‐WT, F‐WT, M‐KO, and F‐KO mice. (B) Linear relationships between the developed tension (*y*‐axis: difference of peak systolic tension and end‐diastolic tension) and end‐diastolic tension (*x*‐axis) in the four groups of mice. Correlation coefficient equals 0.9702 (*P* = 0.0298; M‐WT), 0.9910 (*P* = 0.009; F‐WT), 0.9950 (*P* = 0.005; M‐KO), and 0.9960 (*P *= 0.0038; F‐KO), associated with corresponding slopes of regression lines of 0.09834 ± 0.01737, 0.1570 ± 0.01498, 0.1721 ± 0.01217, and 0.3299 ± 0.02044, respectively. The statistically significant difference in the slope of regression line was presented between M‐KO and F‐KO (*P* = 0.0261), M‐WT versus M‐KO (*P* = 0.02616), F‐WT versus F‐KO (*P* = 0.002358), and M‐WT versus F‐KO (*P* = 0.000978), whereas statistically insignificant between M‐WT and F‐WT mice (*P *= 0.06344). (C) The developed tension curves of the four groups of hearts that were subjected to 14,15‐EEZE for 45 min. Correlation coefficient equals 0.9702 (*P* = 0.0298, M‐WT), 0.9983 (*P* = 0.0017; F‐WT), 0.9571 (*P* = 0.0429; M‐KO), and 0.9939 (*P* = 0.0061; F‐KO), associated with their corresponding slopes of regression lines of 0.09834 ± 0.01737, 0.1065 ± 0.004419, 0.1835 ± 0.0393, and 0.1332 ± 0.01049, respectively. There was no significant difference among all groups (*n* = 5–8 for each group).

Developed tension (*y*‐axis of Fig. 4B and C), represented as a difference between peak systolic and end‐diastolic tension (Fig. 4A) in response to increases in end‐diastolic tension (*x*‐axis) was used as an index of cardiac contractility (Qin et al. 2015b). As summarized in Figure 4B, all groups of hearts displayed dose‐dependent increases in developed tension. Generally, the increment in cardiac contractility was greater in F‐KO > M‐KO = F‐WT > M‐WT mice, respectively, associated with a deeper slope of the regression line in F‐KO than M‐KO mice (*P* = 0.026), as well as a tendency toward a deeper slope in F‐WT than M‐WT mice (*P* = 0.063). Although there was a parallel upward shift in the developed tension curve in M‐KO compared to that of F‐WT mice, their increment at each preload level appeared identical (Fig. 4B), resulting in a comparable slope of the two regression lines (*P* = 0.478). These results coincided with those shown in Figure 1, indicating two issues: (1) disruption of the sEH gene in males (M‐KO) evokes the same cardiac behaviors as those in F‐WT mice, alluding to the presence of female‐specific properties that imitates actions of sEH deficiency in males; and (2) an additional knockout of the sEH gene in females (F‐KO) further promotes cardiac contractility in a “dose‐like”‐dependent manner in comparison with F‐WT and M‐KO mice, revealing an additive/or synergistic effect between the female sex and sEH deficiency.

### Potentiation of cardiac contraction via an EET‐dependent mechanism

Specific contribution of EETs to the enhanced cardiac contractility in F‐WT, F‐KO, and M‐KO mice (Fig. 4B) was evaluated using an EET inhibitor. Since our preliminary study confirmed that there were no effects of 14,15‐EEZE on normal male hearts, we therefore used M‐WT hearts as controls to compare the EEZE‐induced changes in the other three groups of mice. Figure 4C shows that 14,15‐EEZE caused a downward shift in the developed tension curves of F‐WT, F‐KO, and M‐KO mice, returning the originally enhanced cardiac contractility in the three groups of mice back to the comparable levels seen in M‐WT hearts. Consequently, neither the increment in developed tension nor the slope of regression lines was significantly different among the groups, as illustrated by four statistically comparable curves. These data indicate that the difference in cardiac contractility between the sexes (male vs. female) and between different transgenic models (WT vs. KO) was essentially mediated by EETs. Notably, 14,15‐EEZE abolished the significant differences, but failed to completely reverse the developed tension curve in F‐WT and sEH‐KO hearts to the same level as that in M‐WT hearts, due perhaps, to its lower efficiency in inhibiting other isoforms of EETs, including 5,6‐; 8,9‐; or 11,12‐EET, respectively.

**Figure 5:**
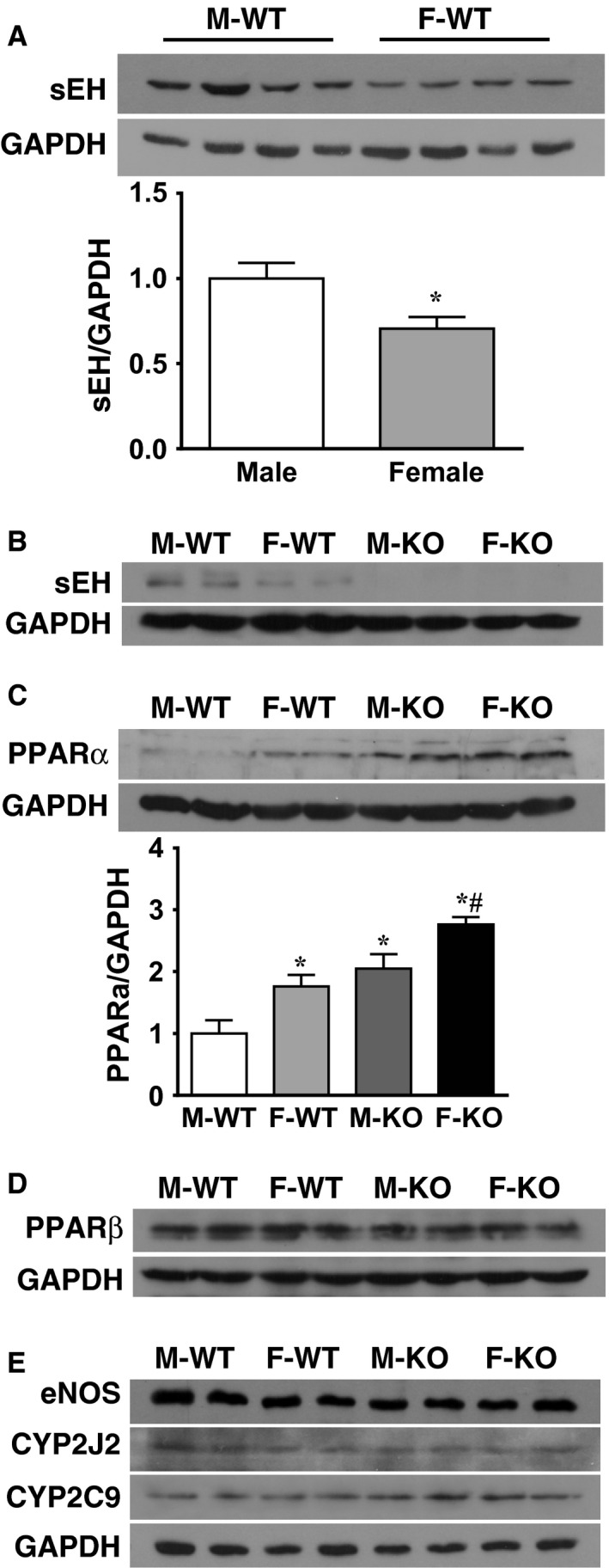
Female‐specific downregulation of sEH and EET‐dependent upregulation of PPAR *α*. Western blot analysis for protein expression of sEH (A and B), PPARs (C and D), and eNOS, CYP2J2, and CYP2C9 (E) in hearts of the four groups of mice. * indicates significant difference from M‐WT. # indicates significant difference from F‐WT and M‐KO (*n* = 3 blots for each summarized data).

Figure 5 provides molecular evidence from western blot analysis. The original and summarized data indicate that protein expression of sEH in myocardial tissue was significantly suppressed in F‐WT compared to M‐WT mice (Fig. 5A) and was undetectable in sEH‐KO mice (Fig. 5B). Protein expression of PPAR*α* (a major subunit of PPAR families expressed in the heart) was significantly increased in myocardium of F‐WT, M‐KO, and F‐KO compared to M‐WT mice; and was significantly greater in F‐KO than F‐WT and M‐KO hearts (Fig. 5C), thus suggesting synergetic effects between female sex and EETs in the promotion of PPAR*α* expression. By contrast, expression of PPAR*β* remained comparable in the four groups of mice (Fig. 5D), further confirming the specificity for PPAR*α* involvement in the pathway. Protein expression of eNOS, CYP2J2 (a major cardiac EET synthase), and CYP2C9 (a major EET synthase of vascular endothelium) was comparable among the four groups of mice (Fig. 5E).

## Discussion

This study demonstrates the following four major findings. First, sEH deficiency increases coronary blood flow that was attributed to the reduced vascular resistance in both basal condition (Fig. 2) and during cardiac work (Figs. 3, 4). Second, cardiac contractility in males and females exhibited different responses when the hearts were challenged with the same increase in preload, characterized as significantly greater responses in F‐WT than M‐WT, and in F‐KO than M‐KO mice, respectively (Fig. 4A and B). Third, blockade of EET bioavailability essentially abolished the differences in cardiac contractility (Fig. 4C), clarifying the nature of EET‐driven responses. Finally, the genetic deletion and downregulation of sEH (Fig. 5A) increased cardiac EETs (Fig. 1), and was accompanied by a secondary upregulation of PPAR*α* expression (Fig. 5C), suggesting PPAR*α* is a downstream mediator of EET‐dependent signaling. As a result, the activation of EETs and subsequent activation of PPAR*α* reciprocally contribute to the adaptation of cardiac performance. Given that the disruption of the sEH gene in males (M‐KO) evokes the same action as downregulation of sEH in F‐WT mice, an additional sEH knockout in the latter (F‐KO) further promotes the response, and that both sexually dimorphic (male vs. female) and transgenic‐dependent (WT vs. KO) differences are prevented by the inhibition of EETs, we therefore draw the conclusion that the reciprocal actions of female sex‐specific and sEH/EET‐mediated signaling converge synergistically in the promotion of cardiac performance, a process that could be amplified by the activation of PPAR signaling.

### sEH deficiency benefits coronary perfusion, as a function of reduced vascular resistance

Under physiological conditions, increases in EETs (Fig. 1) were capable of eliciting a significant decline in vascular resistance (Fig. 3), attributing to the EET‐dependent coronary vasodilator properties and subsequently increasing coronary blood flow (Fig. 2). Particularly, the cardiac EET metabolic pattern (Fig. 1) matched up with the phenotypes of basal coronary flow (Fig. 2) and cardiac performance in working hearts challenged with increases in preload (Figs. 3, 4). Because of the comparable expression of EET synthases (CYP2J2 and CYP2C9; Fig. 5E), the increased cardiac EETs resulted mainly from compromised EET hydrolysis/degradation (Table 1 and Fig. 1). To this end, a physiologically based EET‐dependent adaptation of coronary circulation in normal hearts was clarified, and provides a mechanistic rationale for the clinical development of sEH inhibitors as a therapeutic strategy in the treatment of ischemic heart diseases (Imig 2006; Imig and Hammock 2009). It is worth noting that in the pulmonary circulation, a female‐driven increase in pulmonary EETs did not significantly affect basal right ventricular systolic pressure (RVSP), but did elicit great alterations in RVSP when animals were challenged with thromboxane analog (Kandhi et al. 2015b) or exposed to hypoxia (Kandhi et al. 2015a), insinuating a conditional‐based characteristic of EETs in the pulmonary circulation. In the heart, however, the functional value of EETs is predominantly discernible in both basal and stimulated conditions, suggesting that EETs are vital players in the regulation of coronary circulation.

### Female sex favors sEH deficiency‐dependent adaptation of cardiac function

We found that the reduction in coronary resistance (Fig. 3), increases in coronary flow (Fig. 2), and cardiac contractility (Fig. 4) were inversely correlated with the cardiac sEH expression (Fig. 5A), but were proportional to the expression of PPAR*α* (Fig. 5C). Moreover, during the process of tension development in response to heart stretch, high cardiac EETs in F‐WT and sEH‐KO mice (Fig. 1) served as major players in dilating coronary arteries, which was followed by improved cardiac perfusion. Thus, an optimal utilization of oxygen as a consequence of better tissue perfusion could be responsible for the promotion of cardiac work. Figure 4 clarifies two important issues by showing that (1) during cardiac performance, normal female hearts exhibited a greater potential for tension development than males and (2) the sexual dimorphism in cardiac contractility also exists in sEH null hearts with even greater vigor. This reveals reciprocal interaction between the female‐specific and EET‐dependent potentiation of cardiac function, during which the involvement of PPARs was noticed in the cross talk between the pathways. This conclusion was drawn based on the positive correlations among cardiac work (Fig. 4), EET levels (Fig. 1), and PPAR*α* expression (Fig. 5C), as well as on evidence of the critical roles of PPARs as either downstream targets of EETs or upstream initiators of estrogen/CYP (Imig et al. 2005).

### Mechanisms responsible for the female‐favorable actions of EETs and PPARs

The nature of EET‐dependent contribution to the potentiation of cardiac contractility is indicated in Figure 4C, which shows that the inhibition of EET bioactivity eliminated the differences originally presented in Figure 4B. Female‐specific downregulation of cardiac sEH expression (Fig. 5A) provided explanations for the similar metabolic pattern of EETs and DHETs in F‐WT and sEH‐KO hearts (Fig. 1), bringing to light a functional significance for female‐facilitated actions of EETs (Figs. 2, 3, 4). Even with an ~30% reduction in sEH expression, F‐WT hearts still maintained similar levels of EETs and DHETs to those in sEH‐KO hearts (Fig. 1). This could presumably be explained by the simultaneous presence of reduced sEH activity, in addition to the downregulation of its protein expression. Indeed, previous studies reported that in mouse cerebral arteries, livers, and kidneys, sexually dimorphic changes in sEH gene expression were accompanied by the same change in its enzymatic activity (Pinot et al. 1995; Zhang et al. 2009).

It has been concluded that female sex and/or estrogens favor the contribution of EETs over other vascular mediators including NO (Scotland et al. 2005; Vasudevan et al. 2005). For instance, in the clinically failing human heart, a condition associated with significantly impaired NO synthesis and downregulated PPAR*α* expression (Karbowska et al. 2003), myocyte necrosis and apoptosis were significantly lower in females than in males, leading to a longer duration of the process and a later onset of cardiac decompensation in women (Guerra et al. 1999). These female‐specific cardioprotections are dependent at least in part, upon the EET‐attributable protection against cardiomyocyte necrosis and apoptosis (Oni‐Orisan et al. 2014), as well as EET‐dependent stimulation of PPAR signaling via their actions as PPAR ligands (Kersten et al. 2000). On the other hand, in physiological conditions, the heart derives most of the energy necessary for its contractile function from fatty acid oxidation (Lopaschuk et al. 1994). SEH metabolizes epoxides of fatty acids more rapidly than other substrates (Pinot et al. 1995). As such, in addition to an optimal utilization of oxygen as a function of greater vasodilation‐induced improved tissue perfusion, promoting the uptake of fatty acids such as EETs as a cardiac substrate preserves the energy potential of hearts (Recchia et al. 1999). Since fatty acids function as a primary energy source for cardiac work and their uptake is promoted by PPARs (Goto et al. 2013), and additional increases in EET bioactivities can in turn activate PPAR signaling (Ng et al. 2007), it is plausible to speculate that with downregulation of, or deficiency in sEH in F‐WT and sEH‐KO mice, cardiomyocytes are able to optimally utilize EETs as energy substrates, while the subsequent EET‐dependent activation of PPAR signaling can further lead to vigorous uptake of EETs by the myocardium (Ng et al. 2007; Goto et al. 2013) to form a positive feedback loop. The upregulation of PPAR*α* occurs in not only F‐WT, but also in M‐KO mice as well (Fig. 5C), and additional knockout of the sEH gene in females (F‐KO) elicited further “dose‐like”‐dependent increases in PPAR*α* expression that is accompanied by greater cardiac contractility (Fig. 4) compared with F‐WT and M‐KO mice. These findings, therefore, strongly suggest that PPAR*α* can be a downstream mediator that is triggered by EETs in female‐susceptible manner. As such, a female‐driven potentiation of EETs, associated with consequential activation of downstream located PPAR*α*, works in concert to support ATP synthesis and drive cardiac performance into high gear. Indeed, the functional significance of PPAR*α* has been further highlighted by studies indicating that suppression of PPAR*α* in the failing human heart contributes significantly to the reduction in fatty acid utilization, leading to the formation of “energy starved” hearts (Karbowska et al. 2003), a situation that can be reversed by PPAR*α* agonists via recruitment of myocardial ATP production (Sarma et al. 2012).

**Figure 6:**
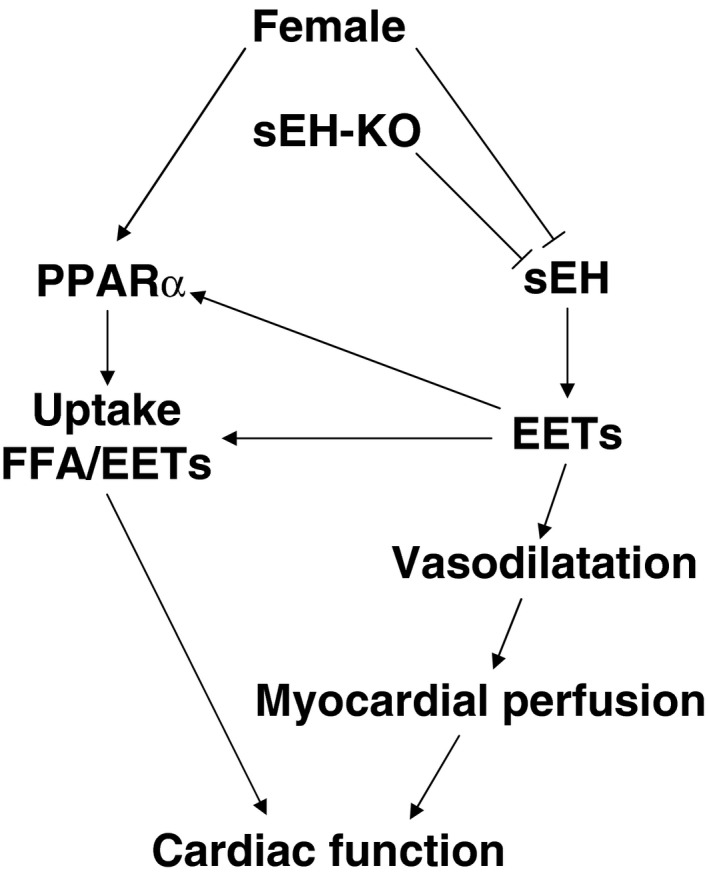
Schematic illustration of the positive feedback loop during the process of EET‐dependent potentiation of cardiac work. Genetic disruption, or female‐specific downregulation of sEH (Fig. 5A) (Meijer et al. 1987; Pinot et al. 1995; Harris and Hammock 2013) and female‐induced upregulation of PPAR *α* (Fig. 5B) (Imig et al. 2005) cause increases in energy substrates of free fatty acids (FFA and EETs, Fig. 1) (Recchia et al. 1999) and their uptake (Lopaschuk et al. 1994). EETs per se in turn, stimulate PPAR *α* expression/activities (Fig. 5B) (Kersten et al. 2000; Imig et al. 2005; Ng et al. 2007) to initiate further vigorous uptake of EETs by the heart (Karbowska et al. 2003; Sarma et al. 2012; Goto et al. 2013). On the other hand, EETs elicit greater vasodilation (Fig. 3) and better cardiac perfusion (Fig. 2), and both synergistically promote contractility of hearts (Fig. 4). ↓ indicates a stimulation; ┴ indicates an inhibition. EET, epoxyeicosatrienoic acid; sEH, soluble epoxide hydrolase; DHETs, dihydroxyeicosatrienoic acids; WT, wild type; KO, knockout; FFA, free fatty acids.

Taken together, the presence of interactions among the sex/gender, sEH expression and EET metabolism, PPAR signaling, and cardiac reactivity have brought us to make the conclusion that there is a female‐favorable adaptation of cardiac function via an EET‐dependent signaling that is amplified by activation of PPARs (Fig. 6).

### Perspectives and alternatives

This study demonstrates that innate female suppression of sEH expression generates a duplicated/overlapping disruption of the sEH gene in the EET‐initiated improvement of cardiac function. This mechanism may explain, at least in part, the “paradox” of the minimal hypotensive effect exhibited in female mice in response to knockout of Ephx2 gene (Luria et al. 2007), and also further supports our previous findings that show an identical phenotype of vascular responsiveness to flow/shear stress (Qin et al. 2015a) and to intravascular pressure (Froogh et al. 2016), as well as the similarities in the pulmonary arterial responses to EETs (Kandhi et al. 2015b) between F‐WT and sEH‐KO mice. Sex differences in the cardiovascular systems have been believed to be intrinsically attributed to sex chromosome‐dependent differences in gene expression, which can be further modified by sex hormones, leading to the sex‐specific gene expression and function (Garcia et al. 2016). To this end, our aim for future studies is to explore molecular mechanisms involving the female hormone/estrogen‐dependent epigenetic regulation of sEH via the DNA methylation‐induced silencing of sEH transcription. On the other hand, while we have shed light on a beneficial modification of PPAR*α* expression as the function of female sex and EETs, its causative role, if any, in the promotion of cardiac contractility needs to be further clarified.

## Conflict of Interest

None.
